# Whole-exome sequencing supports genetic heterogeneity in childhood apraxia of speech

**DOI:** 10.1186/1866-1955-5-29

**Published:** 2013-10-02

**Authors:** Elizabeth A Worthey, Gordana Raca, Jennifer J Laffin, Brandon M Wilk, Jeremy M Harris, Kathy J Jakielski, David P Dimmock, Edythe A Strand, Lawrence D Shriberg

**Affiliations:** 1 Department of Pediatrics, Medical College of Wisconsin, 8701 Watertown Plank Road, Milwaukee, WI, USA; 2 Human and Molecular Genetics Center, Medical College of Wisconsin, 8701 Watertown Plank Road, Milwaukee, WI, USA; 3 Cancer Cytogenetics Laboratory, University of Chicago Medical Center, 5841 S Maryland Avenue, Chicago, IL, 60637, USA; 4 Wisconsin State Laboratory of Hygiene Clinical Genetics Laboratories, 465 Henry Mall, Madison, WI, 53706, USA; 5 Communication Sciences and Disorders, Augustana College, 639 38th Street, Rock Island, IL, 61201, USA; 6 Department of Neurology, Mayo Clinic, 200 First Street, Rochester, MN, 55905, USA; 7 Waisman Center, University of Wisconsin–Madison, 1500 Highland Avenue, Madison, WI, 53705, USA

**Keywords:** Apraxia of speech, Developmental verbal dyspraxia, Next-generation sequencing, Speech disorder, Whole-exome sequencing

## Abstract

**Background:**

Childhood apraxia of speech (CAS) is a rare, severe, persistent pediatric motor speech disorder with associated deficits in sensorimotor, cognitive, language, learning and affective processes. Among other neurogenetic origins, CAS is the disorder segregating with a mutation in *FOXP2* in a widely studied, multigenerational London family. We report the first whole-exome sequencing (WES) findings from a cohort of 10 unrelated participants, ages 3 to 19 years, with well-characterized CAS.

**Methods:**

As part of a larger study of children and youth with motor speech sound disorders, 32 participants were classified as positive for CAS on the basis of a behavioral classification marker using auditory-perceptual and acoustic methods that quantify the competence, precision and stability of a speaker’s speech, prosody and voice. WES of 10 randomly selected participants was completed using the Illumina Genome Analyzer IIx Sequencing System. Image analysis, base calling, demultiplexing, read mapping, and variant calling were performed using Illumina software. Software developed in-house was used for variant annotation, prioritization and interpretation to identify those variants likely to be deleterious to neurodevelopmental substrates of speech-language development.

**Results:**

Among potentially deleterious variants, clinically reportable findings of interest occurred on a total of five chromosomes (Chr3, Chr6, Chr7, Chr9 and Chr17), which included six genes either strongly associated with CAS (*FOXP1* and *CNTNAP2*) or associated with disorders with phenotypes overlapping CAS (*ATP13A4*, *CNTNAP1*, *KIAA0319* and *SETX*). A total of 8 (80%) of the 10 participants had clinically reportable variants in one or two of the six genes, with variants in *ATP13A4*, *KIAA0319* and *CNTNAP2* being the most prevalent.

**Conclusions:**

Similar to the results reported in emerging WES studies of other complex neurodevelopmental disorders, our findings from this first WES study of CAS are interpreted as support for heterogeneous genetic origins of this pediatric motor speech disorder with multiple genes, pathways and complex interactions. We also submit that our findings illustrate the potential use of WES for both gene identification and case-by-case clinical diagnostics in pediatric motor speech disorders.

## Background

Next-generation sequencing continues to transform genomic research and clinical practice. Reductions in the time and cost required to sequence and analyze an individual’s genome or exome (the exonic subset of the genome) have catalyzed descriptive, explanatory research in genomic variations underlying monogenetic and multigenetic diseases and disorders [1-7]. Interpretation of genetic findings in a clinical setting is scarcely a new challenge, but the complexity of this task is increased in next-generation sequencing because of the dramatic increase in dataset size. In the present study, we sought to determine whether whole-exome sequencing (WES) of a small number of individuals could be used to identify variants in candidate or novel genes that may be causally associated with a rare, severe, persistent motor speech disorder termed *childhood apraxia of speech* (CAS).

### Childhood apraxia of speech

CAS, also termed *developmental verbal dyspraxia* in medical and international contexts, is "a neurological childhood (pediatric) speech sound disorder in which the precision and consistency of movements underlying speech are impaired in the absence of neuromuscular deficits (e.g., abnormal reflexes, abnormal tone)" [8]. As in other apraxias (for example, limb apraxia and nonverbal oral apraxia), only volitional (willful, purposeful) movement patterns are affected. The core speech-processing impairment in CAS is in transcoding linguistic representations into the plans for movements that produce articulate speech [9,10]. CAS can occur congenitally or be acquired anytime during the period of speech acquisition, nominally from birth to age nine years. Both onsets of CAS can occur idiopathically, in the context of a number of complex neurodevelopmental disorders or in association with a neurological event [11]. The severity of an individual’s apraxia of speech throughout the lifespan can range from mild to severe, with more recent reviews indicating that individuals may normalize speech but not all prosodic features [8,12]. A central question is whether neurocognitive and neuromotor processing deficits in CAS are substantially similar to those described in adult-onset apraxia of speech, which most frequently is consequent to stroke [13,14] but also occurs as a primary progressive disorder [15]. In the following sections, we review the findings reported in the literature for variants and genes associated with CAS.

### Childhood apraxia of speech and *FOXP2*

The first variant causally associated with CAS was a heterozygous missense mutation in the *FOXP2* forkhead box gene segregating with non–word repetition task deficits and orofacial apraxia in half of the members of a multigenerational family ("KE") [16]. *FOXP2* codes for a protein from the FOX family of winged helix/forkhead transcription factors. It is expressed widely in the fetal and adult brain, where it regulates the expression of other genes within and among cortical, basal ganglia and cerebellar circuits [17,18]. The expression pattern is specific to defined neuronal subpopulations in these different structures (for example, deep layers of the cortex, medium spiny neurons of the striatum and Purkinje cells in the cerebellum). Deficits in these regions during embryogenesis and/or postnatal development are risk factors for early and persistent speech-language disorder and associated cognitive, sensorimotor, learning and affective deficits [19,20].

In addition to other reports of participants with *FOXP2* disruptions, some to all of whom had suspected CAS [21-24], several recent reports using contemporary inclusion criteria to identify CAS and other motor speech disorders have replicated and extended the speech-language and neurocognitive phenotype associated with *FOXP2*. A two-part study series of a mother and daughter with CAS and a breakpoint in a balanced 7;13 translocation disrupting *FOXP2* reported speech profiles consistent with both apraxia and dysarthria [20,25]. Recent neuroimaging studies of affected KE family members have also reported speech and neural substrates consistent with apraxia and dysarthria [26]. A report of a mother and son with CAS and a submicroscopic deletion of *FOXP2*[19] also described apraxia, dysarthria and cognitive language profiles similar to those in the KE family and the mother-and-daughter studies [20,25]. In another report, investigators described a small *de novo* intragenic deletion of *FOXP2* presumed to be causal for a severe motor speech disorder as well as for language and literacy deficits [27]. Behavioral findings in these studies support an emerging view that, in addition to the signature motor speech deficits of CAS and dysarthria, *FOXP2* disruptions are associated with deficits in attentional, auditory-perceptual, sensorimotor, cognitive-linguistic and affective processes [14,28]. In several studies in which suspected CAS and large-scale deletions that affect *FOXP2* were reported, the large number of genes affected raises the possibility of modifier gene effects [22,24]. To date, in all cases in which smaller *FOXP2* variants were associated with CAS, the variants were found in a heterozygous state [16,21,29,30].

A widely cited genetic study of 49 speakers (from several countries) suspected to have CAS identified only one participant (and two of his nuclear family members) with a mutation in *FOXP2*[30]. Findings from this study and unpublished estimates of the attributable risk of *FOXP2* for CAS have been interpreted to indicate that the risk is low (approximately 2%). The constraint on this interpretation is the high rates worldwide of false-positive diagnoses of CAS, which are estimated to be as high as 80% in the most comprehensive literature reviews currently available [8,12], thus suggesting the likelihood of a high percentage of false-positives in the previously referenced clinical sample of children suspected to have CAS [30]. In a recent report of 24 participants whose suspected CAS was confirmed using a standardized classification procedure, one participant was found to have a *FOXP2* mutation [29], effectively increasing this clinical estimate of the attributable risk for CAS of a *FOXP2* mutation or disruption to approximately 4%.

Reviews of the extensive avian and murine *Foxp2* modeling literature, as well as the considerable literature on the role of this gene in evolutionary biology, are beyond the scope of the present paper. Reviews describe the array of *Foxp2* model findings consistent with abnormal vocal development and performance (for example, disrupted synaptic plasticity in the striatum, cerebellar and radial glial cell abnormalities, abnormal esophageal development and mild to severe motor impairment) (for example, see [31-37]). Homozygous null *Foxp2* mice have cerebellar abnormalities with incompletely folded folia, as well as embryonic, neonatal or perinatal lethality, whereas the heterozygous mutation does not result in premature death but is associated with modest developmental delay, abnormal motor learning and a significant alteration in ultrasonic vocalization in response to parental separation (for example, see [38-40]).

### Childhood apraxia of speech, *CNTNAP2* and *FOXP1*

A number of additional candidate genes and variants have been described in the emerging CAS genetics literature and associated verbal traits and disorders literature. The widely studied *CNTNAP2* gene encodes a member of the neurexin family, which functions in the vertebrate nervous system as cell adhesion molecules and receptors [41,42]. *CNTNAP2* has been implicated in intellectual disability [43,44], cortical dysplasia-focal epilepsy syndrome and the related Pitt-Hopkins-like intellectual disability [43], autism [41,45,46] and language impairment [47,48]. A recent review described *CNTNAP2* in the context of neurogenetic networks during development and summarizes the known information on the regulation and function of this gene [49]. Notably, findings implicating *CNTNAP2* in both autism [41] and language impairment without autism [47] suggest distinct *CNTNAP2* pathways for verbal trait disorders, including CAS. In one study, investigators posited that variants in *CNTNAP2* and *FOXP*1 may interact to contribute to the phenotype of an individual [50]. Several useful reviews of genetic findings in verbal trait disorders, including CAS, are available in the literature (for example, see [27,36,51-55]).

### Childhood apraxia of speech in complex neurodevelopmental disorders

We have proposed that, especially in view of the low-point prevalence of idiopathic CAS, estimated at 1 per 1,000 to 1 per 3,000 in 3- to 6-year-old children in the general population [8,12], studying CAS in the context of complex neurodevelopmental disorders provides a rich source of comparative information on genomic pathways [11]. CAS has been reported in association with a number of complex neurodevelopmental disorders, including galactosemia [56], rolandic epilepsy [57-59], 16p11.2 microdeletions [60,61] and Williams-Beuren microduplication [62,63]. Preliminary reports also indicate speech phenotypes consistent with CAS in participants with Down syndrome [9] and 22q11 microdeletion syndrome [64]. Clearly, in addition to the singular finding that disruptions in *FOXP2* are a sufficient (monogenic) cause of CAS, continuing research cited in a later section supports heterogeneous genomic pathways to CAS.

### Statement of purpose

The study reported herein had two goals. The first was to identify, in a sample of well-characterized participants with idiopathic CAS, candidate genomic variants supporting literature trends indicating genetic heterogeneity in CAS. The term *well-characterized* is important because the most significant methodological problem in all studies of speech impairment consistent with CAS, including those in the literature on CAS in participants with *FOXP2* mutations and disruptions, is the lack of consensus on the inclusionary criteria to classify participants as positive for CAS. The second goal was to evaluate the use of next-generation sequencing in the clinical establishment of disease pathogenicity in this and other pediatric motor speech disorders. Both goals are motivated by reports in other contexts of the significant clinical potential of sequencing for an eventual understanding of the genomic substrates of diseases and disorders [65-69].

## Methods

### Participants

Ten participants with the clinical diagnosis of CAS or suspected CAS were enrolled for a study of pediatric motor speech sound disorders. They were recruited and consented using procedures approved by institutional review boards at two collaborative sites. A trained examiner at each site administered the Madison Speech Assessment Protocol (MSAP), a lifespan assessment protocol for diagnostic classification of pediatric speech sound disorders, including CAS [70,71]. A total of 15 speech tasks provide age-sex standardized scores on the competence, precision and stability of a speaker’s speech, prosody and voice. Audiorecordings of the speech samples obtained from all MSAP tasks were processed using computer-aided methods for perceptual and acoustic analyses. Each of the 10 participants met classification criteria for CAS based on their standardized scores on a diagnostic marker of CAS and supplementary documentation with high to conclusive diagnostic accuracy [9].

Table 1 gives the phenotype information for the 10 participants. All participants were self-reported as white; no additional heritage information was collected. Individual phenotype information was obtained from the parent questionnaire, and direct assessment measures are cited in the Table 1 footnotes. To maintain participant anonymity, individual participant data are aggregated in three developmental age groups.

**Table 1:** **Phenotype data for 10 participants with childhood apraxia of speech**^**a**^

**Participants**				**Familial status**^**c**^	**Cognitive impairment**^**d**^	**Language impairment**			**Motor impairment**	
**Participant no.**	**Gender**	**Age group (yr)**^**b**^	**Years of apraxia treatment**			**Onset**^**e**^	**Comprehension**^**f**^	**Expression**^**f**^	**Gross**^**g**^	**Oral nonverbal**^**h**^
1	M	C	7+	+	+	+	+	+	+	+
2	M	A	3	+		+		+	+	ND
3	F	C	6	ND		+	+	+	ND	ND
4	M	C	6			+	+	+		+
5	F	C	7+	+	+	+	+	+	+	ND
6	F	A	5			+	+	+	+	+
7	F	B	4	+		ND			+	+
8	M	B	3		+	+	ND	ND	+	+
9	F	A	4			+	+		+	+
10	M	B	5	+		+	+	+	+	+

^a^Plus signs indicate impairment. Blank cells indicate negative history or performance within normal limits. ND indicates no available data. ^b^Age groups: A = 3 to 6 years; B = 7 to 9 years; C = 10 to 19 years. ^c^One or more nuclear family members with a verbal trait disorder, including speech disorder, language disorder, reading disorder, cognitive disability or learning disability. ^d^Composite IQ <85 [72]. ^e^Late onset of babbling, first word, two words together or short phrases per parent report, ^f^Listening Comprehension and Oral Expression Scales standard scores <85 [73]. ^g^Parent report or history of physical or occupational therapy. ^h^Oral nonverbal motor assessment tasks.

The 10 participants were evenly divided by gender, a sampling outcome not representative of the 70% and higher prevalence of males typically reported in studies of idiopathic CAS [8,12]. Participants include three preschool children ages 3 to 6 years (age group A), three early school–age children ages 7 to 9 years (age group B) and four older participants ages 10 to 19 years (age group C). The number of years of treatment for the oldest participants documents the persistence of CAS. For many to most individuals with CAS, residual speech-language deficits persist over the lifespan, frequently including residual speech errors, inappropriate pausing, inappropriate linguistic stress and slow speech rate [9].

Familial aggregation was considered to be positive if one or more of the participant’s nuclear family members had a history of any type of speech sound disorder, adjusted for missing data. The 56% familial aggregation rate in Table 1 is consistent with familial aggregation estimates for convenience samples of children with the most common form of speech sound disorder, termed speech delay [74], supporting the likelihood that at least some forms of both idiopathic speech delay and idiopathic CAS are inherited. The design of the present study did not allow for follow-up segregation analyses. Although a history of other forms of speech disorders was present in family members, the case history information indicated that none of the 10 participants had a nuclear family member with a clinical diagnosis of CAS.

The profiles of cognitive, language and motor impairment characteristics of the 10 participants with CAS were similar to those summarized in the two most recent comprehensive reviews of CAS research [8,12]. Profiles were also similar to behavioral data in these domains for affected family members in three published reports of families with *FOXP2* mutations [19]. Adjusted for missing data, 30% of this sample of 10 participants with idiopathic CAS met assessment criteria for cognitive disability, 100% were delayed in the onset of speech and language, and 78% had active, persistent impairments in either or both language comprehension and language expression. Last, 89% and 100% of participants, respectively, had some level of impairment in gross motor and oral nonverbal movements. As discussed elsewhere and cited previously, these behavioral profiles of CAS are consistent with emerging perspectives on CAS as a multiple-domain disorder involving deficits in auditory-perceptual encoding and memory processes in addition to the signature motor speech deficit [14].

### *FOXP2* sequencing

All 10 participants were evaluated for *FOXP2* mutation status by sequencing each of the seventeen *FOXP2* coding exons [NCBI:NM_014491.3]. The exons were amplified by polymerase chain reaction (PCR) (AmpliTaq Gold PCR Master Mix; Applied Biosystems, Carlsbad, CA, USA) using oligos referenced elsewhere [29]. PCR amplification and amplicon size were verified by gel electrophoresis. Sequences of each PCR amplicon were generated in both forward and reverse direction sequencing reactions using the BigDye Terminator v3.1 Cycle Sequencing Kit (Applied Biosystems), purified with AxyPrep Mag DyeClean beads (Axygen Biosciences, Union City, CA, USA) and run using either an ABI 3730xl or 3130xl DNA Analyzer (Applied Biosystems). No *FOXP2* variants of note were identified.

### Whole-exome sequencing: primary and secondary analysis

We completed WES for each participant by subjecting whole-blood–derived genomic DNA to in-solution hybrid capture and Illumina sequencing. Library preparation, sequencing, read mapping and variant calling were performed at the University of Wisconsin Biotechnology Center DNA Sequencing Facility using the Genomic DNA Sample Prep Kit (Illumina, Inc, San Diego, CA, USA) with the following modification to the manufacturer’s protocol: DNA was sheared using the Bioruptor XL (Diagenode sa, Liege, Belgium) for 30 minutes using a cycle of 30 seconds on followed by 30 seconds off on high power. After adapter ligation, samples were size-selected to an average size of 300 ± 50 bp using an Invitrogen E-Gel SizeSelect 2% Agarose Gel (Life Technologies, Grand Island, NY, USA). Solution hybrid capture was performed on the completed libraries using the Agilent SureSelect Human All Exon Capture System (Agilent Technologies, Santa Clara, CA, USA), either 38 Mb (participants 3, 5, 6, 7 and 8) or 50 Mb (participants 1, 2, 4, 9 and 10) with minimal modification of the manufacturer’s protocol.

The finished libraries were quantified using PicoGreen fluorometric dye (Life Technologies), and quality was assessed using the Agilent Bioanalyzer with DNA 1000 Kit chips (Agilent Technologies). Completed libraries were sequenced using the Illumina Genome Analyzer IIx Sequencing System (paired-end 75-bp reads). Sequencing produced between 23 million and 45 million passing filter reads per library. Reads were mapped to the human reference genome assembly, and variant calling (for substitutions, insertions and deletions) was performed using Illumina Consensus Assessment of Sequence and Variation (CASAVA) software. Because of the noted lack of maturity of the available exome-based structural variant calling algorithms [75], we did not undertake calling of this class of variants. Table 2 provides information on the sequencing runs and output. Although there is variation, exome sequencing for all samples was carried out to sufficient depth to differentiate sequencing or mapping errors from heterozygous or homozygous variants. An average of 116,836 high-quality variants were called per sample.

**Table 2:** Sequencing run statistics

**Participants**	**Exon capture**	**No. of lanes**	**No. of total reads passing filter**	**Average coverage (80% on target)**
1	50 Mb	1	39,188,279	94X
2	50 Mb	1	40,950,124	98X
3	38 Mb	1	15,076,800	48X
4	50 Mb	1	45,037,091	108X
5	38 Mb	2	24,806,520	78X
6	38 Mb	2	20,198,040	64X
7	38 Mb	1	26,815,926	84X
8	38 Mb	2	35,035,920	110X
9	50 Mb	1	42,500,956	102X
10	50 Mb	1	23,439,839	56X

### Tertiary analysis

Tertiary analysis aimed at identifying candidate disease-causing variants was performed using CarpeNovo, a tertiary sequencing analysis software platform developed to support the Genomic Medicine Clinic and Program at Medical College of Wisconsin/Children’s Hospital of Wisconsin (MCW/CHW). This Java- and Oracle-based platform has been used at MCW/CHW to identify causative mutations in a variety of disorders (for example, see [67,69]) and has undergone the rigorous testing and validation required for use in this clinical setting. Upward of 100 annotations were tagged to each of the WES-identified variants. CarpeNovo was subsequently used to prioritize some and deprioritize other variants to produce a targeted subset of compelling variants within known or novel candidate genes. Specifically, we prioritized the variants guided by the six assumptions outlined in the paragraphs below.

First, because CAS is a rare disorder and mutations reported to be associated with the phenotype or overlapping phenotypes (for example, autism) have also been novel or rare, we expected the candidate variants to have a low allele frequency within control populations. We therefore deprioritized found variants that have an allele frequency greater than 0.3% in the Single-Nucleotide Polymorphism Database, build 137; the National Heart, Lung, and Blood Institute Exome Sequencing Project 6500 dataset obtained via the Exome Variant Server ftp site; the 1000 Genomes Project variant dataset available from their download site; or our in-house–derived dataset of 128 exomes or genomes.

Second, we prioritized variants based on their likelihood of being deleterious to the normal functioning of the genomic feature, following the American College of Medical Genetics and Genomics (ACMG) guidelines on classification of variants (outlined in Table 3). Specifically, variants were prioritized into three classes: (1) variants already shown to impact function of the underlying genomic feature (for example, transcript, binding site and promoter); (2) variants of a type likely to impact the function of the underlying feature, that is, premature stop causing, start codon altering, canonical splice site altering, read-throughs of the stop codon and frame shift causing insertions or deletions; and (3) variants of a type that may or may not alter the function of the feature, that is, near–splice site variants (within 10 bases of splice sites), in-frame insertions and deletions within protein-coding regions and protein-coding nonsynonymous (NS) changes that were predicted to be damaging by algorithms designed to predict the effect of such changes, that is, PROVEAN (Protein Variation Effect Analyzer), SIFT (Scale Invariant Feature Transform), Mutation Assessor, Condel and PolyPhen-2. The remaining variants either could not be prioritized on impact owing to insufficient data or would not be prioritized because they were unlikely to cause an effect. Variants that, upon further analysis, were shown to be undeniably associated with a phenotype with no conceivable overlap with CAS (based on expert interpretation) were also excluded from further consideration.

**Table 3:** **Summary data on variants by classification status**^**a**^

	**Average gene count**	**Gene range**	**Average variant count**	**Variant range**
Highly likely deleterious and rare (<0.03)	49.40	(44 to 54)	59.20	(53 to 66)
Rare (<0.03) and premature stop	17.40	(16 to 27)	21.80	(19 to 27)
Rare (<0.03) and read-through	0.25	(0 to 1)	0.40	(0 to 1)
Rare (<0.03) and start codon changing	0.00	(0 to 1)	0.60	(0 to 1)
Rare (<0.03) and splice site	6.40	(3 to 8)	6.40	(3 to 8)
Rare (<0.03) and frame shift insertion	15.40	(12 to 20)	17.60	(13 to 22)
Rare (<0.03) and frame shift deletion	11.80	(6 to 17)	12.00	(6 to 17)
Possibly deleterious and rare (<0.03)	207.00	(181 to 219)	239.80	(238 to 249)
Damaging HumVar PolyPhen NS change	114.60	(105 to 127)	137.20	(120 to 152)
Damaging SIFT NS change	96.00	(78 to 111)	130.40	(111 to 153)
Protein coding in-frame insertion	2.00	(0 to 6)	2.00	(0 to 6)
Protein coding in-frame deletion	4.20	(0 to 6)	4.20	(0 to 6)
Intronic near-splice	86.20	(76 to 84)	91.80	(78 to 101)
Any clinical association and rare (≤0.03)	11.80	(1 to 16)	12.60	(1 to 14)
Prioritized clinically associated with CAS but not rare	0.40	(0 to 1)	0.40	(0 to 1)
Prioritized clinically associated with CAS and rare (<0.03)	0.40	(0 to 1)	0.40	(0 to 1)

^a^CAS, childhood apraxia of speech; NS, nonsynonymous; SIFT, Scale Invariant Feature Transform.

Third, to exclude sequencing or mapping errors, we deprioritized variants present in fewer than 10% of reads at that position.

Fourth, we considered variants supporting an autosomal-recessive mode of inheritance and variants exerting their effects in a dominant fashion. Although we did not place a specific filter on zygosity, we did hypothesize that (1) a variant giving rise to a single defective allele may well be sufficient to alter speech and language development while leaving other gene functions intact [23,32,35], and (2) a homozygous variant or compound heterozygote would lead to a more severe or different phenotype.

Fifth, given the existing CAS associations with *FOXP1*, *FOXP2* and *CNTNAP2*, we postulated that we might not be looking for a single gene, but rather for variants in multiple genes giving rise to speech production defects within neurologic networks. All variants within known CAS genes were reviewed, as were variants in genes that had been identified as genes of interest in another sample. Attempts to require that the candidate variants or genes be shared across all or the majority of samples were not fruitful.

Sixth, we excluded variants previously confirmed, in-house or externally, to be neutral polymorphisms.

Following prioritization, 401 variants on average per sample remained under consideration (about 0.34%). In some cases, variants that did not meet these criteria were reviewed, including those within known CAS genes or genes associated with a phenotype with a potential genetic overlap with CAS (including dyslexia and other types of speech impairment) or in genes that had been identified as being of interest in another sample. Each variant was considered based on variant-specific and feature-level functional data. A variety of automated data sources (the Human Genome Mutation Database and the Online Mendelian Inheritance in Man database) and manually accessed data sources (Ingenuity Systems IPA pathway analysis tool (Ingenuity Systems, Redwood City, CA, USA), the GeneCards compendium ( http://www.genecards.org/), the Rat Genome Database ( http://rgd.mcw.edu/), the Mouse Genome Informatics database ( http://www.informatics.jax.org/) and PubMed ( http://www.ncbi.nlm.nih.gov/pubmed/)) were used to examine the function for each candidate. Pathway analyses were also performed to determine whether any of the candidates shared pathways of interest for this disorder. For each variant, the variant impact and the functional association were analyzed in unison to identify pairings resulting in reportable findings (that is, where there was a likely impact and the function was already or could be associated with CAS). The process was performed for each of the 10 samples to identify variants potentially associated with altered neural substrates for verbal traits.

### Sanger validation

All candidate variants that met evidence thresholds for clinical reporting were validated. When available, we used resequencing amplicons (RSA) primers that have been previously designed to cover coding regions in a selection of genes. For regions that did not have RSA primers available, we designed primers using PrimerQuest Software (Integrated DNA Technologies, Coralville, IA, USA). We performed PCR using 10 ng of DNA and performed Sanger capillary sequencing of the PCR product using forward and reverse primers on an ABI 3130xl Genetic Analyzer.

## Results and discussion

For 8 (80%) of the 10 participants, the methods above identified likely pathogenic variants or variants of uncertain significance (VUS) in genes that have previously been shown to be causally associated with CAS or closely related phenotypes. In other participants, we identified likely pathogenic variants in genes of uncertain significance (GUS). To be clear, we used an annotation of VUS in the same way that it is used in a diagnostic laboratory according to the ACMG guidelines. This definition indicates that we believe the variants to be excellent candidates for causation of CAS in these participants. VUS and GUS findings will require follow-up studies to determine whether associations reach levels required for clinical support, either through development of functional assays or by confirmation of the association through identification of causative variants in additional unrelated individuals with CAS. In two participants, we did not identify any reportable findings. Table 4 and Table 5 provide a summary of these highest-priority variants identified in each of the participants for each of the 10 participants. With regard to the two goals of this study, we report not only the pathogenic/likely pathogenic variants but also the VUS and GUS findings that could be pathogenic with external confirmation. The following discussion is ordered by findings for six genes.

**Table 4:** **Summary of most highly prioritized variants in each study participant**^**a**^

**Participants**	**Genes**	**Chromosomes**	**Coordinates**	**EVS allele frequency**	**TGP allele frequency**	**dbSNP ID**	**Zygosity**	**Reads supporting call (%)**
1	*ATP13A4*	Chr3	193,171,979-193,171,979	0.0886	0.05576	rs35424709	Heterozygous	60.47
	*KIAA0319*	Chr6	24,588,884-24,588,884	0.427	0.234	rs4504469	Heterozygous	52.08
2	*ATP13A4*	Chr3	193,171,979-193,171,979	0.0886	0.05576	rs35424709	Heterozygous	45.76
3	No reportable findings							
4	*CNTNAP2*	Chr7	148,106,475 to 148,106,477	Not found	Not found	Not found	Heterozygous	74.36
	*ATP13A4*	Chr3	193,171,979-193,171,979	0.0886	0.05576	rs35424709	Heterozygous	53.97
5	No reportable findings							
6	*CNTNAP2*	Chr7	146,372,040-146,372,040	Not found	Not found	Not found	Heterozygous	38.46
7	*CNTNAP1*	Chr17	38,101,263-38,101,263	Not found	Not found	Not found	Heterozygous	40
8	*FOXP1*	Chr3	71,185,577-71,185,577	Not found	Not found	Not found	Heterozygous	93.33
9	*KIAA0319*	Chr6	24,588,884-24,588,884	0.427	0.234	rs4504469	Heterozygous	41.96
	*SETX*	Chr9	135,204,010-135,204,010	0.0009	0.0096	Not found	Heterozygous	48.17
10	*CNTNAP2*	Chr7	148,106,475 to 148,106,477	Not found	Not found	Not found	Heterozygous	74.36
	*KIAA0319*	Chr6	24,588,884-24,588,884	0.427	0.234	rs4504469	Heterozygous	57.5

^a^dbSNP, Single-Nucleotide Polymorphism Database; EVS, Exome Variant Server; TGP, 1000 Genomes Project.

**Table 5:** **Summary of most highly prioritized variants in each study participant**^**a**^

**Participants**	**Genes**	**Total depth coverage**	**Impact**	**Nucleotide description**	**Protein position**	**Associated phenotype**	**Functional association**	**Report category**
1	*ATP13A4*	43	NS damaging	g.1938A>T	p.Glu646Asp	ASD, CAS	Postulated roles in the developing nervous system and early neuronal development	VUS
	*KIAA0319*	96	NS damaging	c.931G>A	p.Ala311Thr	Developmental dyslexia, SLI	Adhesion between migrating neurons radial glial fibers	VUS
2	*ATP13A4*	59	NS damaging	g.1938A>T	p.Glu646Asp	ASD, CAS	Postulated roles in the developing nervous system and early neuronal development	VUS
3	No reportable findings							
4	*CNTNAP2*	39	Splice consensus	c.3714-7insTTG	N/A	Intellectual delay, ASD, CAS	Local differentiation of axons into distinct functional subdomains	VUS
	*ATP13A4*	63	NS damaging	g.1938A>T	p.Glu646Asp	ASD, CAS	Postulated roles in the developing nervous system and early neuronal development	VUS
5	No reportable findings							
6	*CNTNAP2*	52	NS damaging	c.511C>T	p.Arg171Cys	Intellectual delay, ASD, CAS	Local differentiation of axons into distinct functional subdomains	Likely pathogenic
7	*CNTNAP1*	10	NS damaging	c.3191G>A	p.Arg1064Gln	No human phenotype	Formation and maintenance of neuronal cell connections	VUS in GUS
8	*FOXP1*	15	NS damaging	c.320T>C	p.Ile107Thr	Developmental delay, expressive language deficits, ASD	Regulation of gene transcription during development	Likely pathogenic
9	*KIAA0319*	112	NS damaging	c.931G>A	p.Ala311Thr	Developmental dyslexia, SLI	Adhesion between migrating neurons and radial glial fibers	VUS
	*SETX*	191	NS damaging	g.2975A>G	p.Lys992Arg	AOA2	Suggested to be involved with DNA and RNA processing	VUS in GUS
10	*CNTNAP2*	39	Splice consensus	c.3714-7insTTG	N/A	Intellectual delay, ASD, CAS	Local differentiation of axons into distinct functional subdomains	VUS
	*KIAA0319*	80	NS Damaging	c.931G>A	p.Ala311Thr	Developmental dyslexia, SLI	Adhesion between migrating neurons and radial glial fibers	VUS

^a^AOA2, Ataxia and oculomotor apraxia type 2; ASD, Autism spectrum disorder; CAS, Childhood apraxia of speech; GUS, genes of uncertain significance; NS, nonsynonymous; SLI, specific language impairment; VUS, variants of uncertain significance.

### FOXP1

Participant 8 has a heterozygous novel variant yielding a NS amino acid change in *FOXP1* (p.Ile107Thr) predicted to be damaging by PROVEAN, SIFT, Mutation Assessor, Condel and PolyPhen-2. Isoleucine at this position in the amino acid sequence is absolutely conserved down to fish and lies in a highly conserved region of the protein.

*FOXP1* is expressed in particular neuronal subpopulations in the developing brain [76-78], but its precise roles in brain development have yet to be defined. In animal models, homozygous loss of *FOXP1* causes embryonic, neonatal or perinatal lethality with abnormal cardiovascular system development, including outflow tract septation, ventricular septal defects, abnormal cardiac valve morphology, decreased and irregular heart rate, disorganized myocardium, thin ventricular compact zone and edema [79]. In other studies, homozygous *FOXP1* knockouts have shown alterations in development of motor neurons in the spinal cord [76,80,81]. In contrast, mice with heterozygous *FOXP1* disruption develop normally with regard to their cardiovascular system [79].

*FOXP1* is known to have a global impact on neural development and has been associated with CAS in a number of reports [82]. Another study [83] reported two *de novo* heterozygous mutations (one small deletion and R525X) in the *FOXP1* gene in two unrelated children with moderate intellectual disability, expressive language deficits and autistic features. WES was recently used to identify variants in *FOXP1* causing a frame shift and premature stop codon in an individual with intellectual disability, severe autism spectrum disorder and delayed language [50].

In another study, a heterozygous 785-kb deletion in 3p14.1 that affected *FOXP1*, *EIF4E3*, *PROK2* and *GPR27* was identified in a participant with speech delay, contractures, hypertonia and blepharophimosis [59]. The effect was attributed to *FOXP1*, but the effects of *PROK2* and *GPR27* are unclear, particularly given their known role in developmental delay [59]. Additional support for the effects of *FOXP1* in this case is provided by a study that identified a 1-Mb interstitial 3p14.1 microdeletion including the entire coding region of *FOXP1* in an adult with a recognizable phenotype of autism, motor incoordination, dysmorphic features and severe speech delay [84]. Another study provided additional evidence of involvement due to a deletion exclusively affecting the *FOXP1* gene in a child with motor development delay and impaired language acquisition [57]. It was noted that the presence of a Chiari malformation type I (cerebellar tonsil abnormality) in this individual could also have caused or contributed to motor and speech development delay. Large *de novo* heterozygous deletions affecting *FOXP1* and other genes were also found in three individuals with moderate intellectual disability, gross motor delay and severe speech and language impairment characterized by delayed onset of speech, dysgrammatism and poor speech articulation [58]. A *de novo* intragenic *FOXP1* deletion was also seen in an individual with both intellectual disability and severe language impairment, particularly regarding expressive language [83].

Together, the findings from these studies provide compelling evidence that disruptions in the *FOXP1* gene can be causative of multiple neurodevelopmental abnormalities, including specific language impairment (SLI). Thus, although the variant in the present paper has not previously been reported, it is of a type that would be expected to alter function and was found in a gene shown to be causally associated with the same or a related clinical phenotype. The participant has only a single heterozygous variant, but that has been shown to be sufficient for disease. This individual has the cognitive impairments and gross and oral nonverbal motor impairment seen in many other individuals with *FOXP1*-associated CAS (Table 1). This variant reaches required levels of evidence and is reported as a likely pathogenic variant.

### CNTNAP2

Participant 6 was found to have a heterozygous novel variant giving rise to a NS amino acid change in the *CNTNAP2* gene (p.arg171Cys) predicted to be damaging (by PROVEAN, SIFT, Condel, Mutation Assessor and PolyPhen-2). The variant has not previously been reported but is of a type that would be predicted to alter appropriate functioning of the gene.

The *CNTNAP2* gene is widely studied in verbal trait disorders [41,42]. It is expressed at high levels in the prefrontal and anterior temporal cortex, as well as in the dorsal thalamus, caudate, putamen and amygdala [85]. The protein is localized at the juxtaparanodes of myelinated axons, is associated with potassium channels and has been postulated to play a role in the local differentiation of the axon into distinct functional subdomains. Homozygous mouse models of *CNTNAP2* show abnormalities in the central nervous system, leading to abnormal neuronal migration, corpus callosum structure and interneuron morphology, in addition to abnormal spatial learning, abnormal coordination and interaction behavior, including abnormal vocalization.

*CNTNAP2* has been associated with intellectual disability [43,44]; language delays in children with autism [45]; the language deficits and repetitive, stereotyped behavior in autism [41,46]; and SLI without autism spectrum disorder [47,48]. Large-scale genotyping studies of *CNTNAP2* have been completed with participants who have deficits in common forms of SLI [47] and with delays in the age of the first spoken word in participants with autism [48]. Deletions of this gene have been associated with speech and mild motor delay in a single male participant, although this was complicated by a milder phenotype in a sibling sharing the same deletion [86]. Another speech study identified deletions in this gene in one child with CAS [29]. The identified deletion maps within the noncoding portion of the gene (intron 13) but may affect regulatory elements important for appropriate expression. Given the reports supporting causative associations between *CNTNAP2* and CAS, this variant reaches required levels of evidence for clinical reporting as a likely pathogenic variant in CAS.

Participants 4 and 10 both share a novel, interesting variant in *CNTNAP2*: a heterozygous insertion of three nucleotides directly neighboring the splice site (within the splice consensus sequence at position -7). Heterozygous splice site variants in this gene have previously been associated with autism spectrum disorders and developmental language impairment [43]. Those investigators suggested that a milder phenotype is associated with heterozygous variants, whereas homozygous or compound heterozygous defects are associated with severe intellectual disability, including cortical dysplasia-focal epilepsy syndrome and the related Pitt-Hopkins-like mental retardation. Because of insufficient evidence on the deleterious nature of this variant, the near-splice insertion in participants 4 and 10 is reported as a VUS in a gene strongly associated with CAS.

### CNTNAP1

Participant 7 has a novel heterozygous variant giving rise to a NS amino acid change in *CNTNAP1* (p.Arg1064Gln, c.3191G>A) predicted to be damaging (by PROVEAN, SIFT, Condel, Mutation Assessor and PolyPhen-2). The variant has not previously been reported but is of a type that may alter function.

CNTNAP1 protein is transcribed predominantly in brain tissue and associates with the contactin-PTPRZ1 complex, which is known to play a critical role in the formation and maintenance of neuronal cell connections [87-89]. The CNTNAP1 extracellular domain architecture has been postulated to be the signaling subunit of contactin, enabling recruitment and activation of intracellular signaling pathways in neurons. Neurodegenerative phenotypes, including impaired balance, paresis, hypoactivity and ataxia and embryonic, neonatal or perinatal lethality are seen in homozygous *CNTNAP1* mouse models [90,91]. Abnormal axon morphology, hypermyelination and nerve conduction are present. A milder, nonlethal phenotype (albeit with ataxia and abnormal motor coordination), without the serious morphological alterations seen in homozygotes, is seen in heterozygous mice.

We hypothesize that this heterozygous variant in *CNTNAP1* may be deleterious in participant 7, but this finding does not reach required levels of evidence for clinical reporting. For CAS, we consider *CNTNAP1* a GUS. Confirmation of the role of *CNTNAP1* in CAS will require additional studies of the type illustrated in this report.

### SETX

Participant 9 has a novel heterozygous variant giving rise to a NS amino acid change in *SETX* (p.Lys992Arg, g.2975A>G) predicted to be damaging (by PROVEAN, SIFT, Condel, Mutation Assessor, and PolyPhen-2). The variant has previously been reported in *cis* with *H2197R* in a participant with ataxia and oculomotor apraxia type 2 (AOA2). AOA2 is a neurodegenerative disorder characterized by elevated levels of serum α-fetoprotein, cerebellar atrophy, gait ataxia, peripheral sensorimotor neuropathy, areflexia, saccadic ocular pursuit with nystagmus and variable oculomotor apraxia. Onset ranges from childhood through early adulthood, with the majority of cases arising during adolescence. Senataxin protein is produced in a wide range of tissues, including the brain, spinal cord and muscles. This variant is found in the DNA/RNA helicase domain of the protein. Dysarthria has been seen in all published cases of AOA2, including in participants with the previously reported p.Lys992Arg variant [92]. Both exonic/multiexonic deletions and duplications that disrupt *SETX* leading to AOA2 have been reported [93-95]. AOA2 is inherited in an autosomal-recessive manner [93,94]. This variant is reported as a VUS in a gene previously associated with an overlapping phenotype.

### KIAA0319

Participant 1 has a variant of interest in the *KIAA0319* gene. The variant (p.Ala311Thr, c.931G>A) is believed to be damaging, having been associated with dyslexia and SLI in multiple unrelated families [96,97]. This variant was deprioritized in the first analytic focusing on rare alleles due to an allele frequency that was higher than the allele frequency threshold applied. The variant was, however, noted to be of interest in our analysis of predicted deleterious variants previously associated with potentially associated phenotype, where we place less weight on the currently predicted allele frequency.

*KIAA0319* has a role in appropriate adhesion between migrating neurons and radial glial fibers and may also regulate growth and differentiation of dendrites in the developing brain [97-99]. The gene has four polycystic kidney disease domains with homology to the extracellular domains of the PKD protein PKD1, which are involved in cell-adhesive functions [100]. Both tissue-specific and temporal expression patterns in the frontal and cerebral neocortex, ganglionic eminence, mesencephalon, hippocampus and dentate gyrus and in the Purkinje cell layer of the cerebellum correspond well with the known process, timing and localization of neuronal migration [97]. RNA interference (RNAi) of *KIAA0319* in rats was shown to disrupt neuronal migration and taken as evidence of a role for *KIAA0319* in neuronal migration during formation of the cerebral cortex [101].

Using linkage and association analyses, *KIAA0319* has been shown to have a strong association with language impairment phenotypes, including articulation, text reading and general language skills [97,102,103]. In one study, investigators found strong associations of three single-nucleotide variants (SNVs) with altered speech, language and reading phenotypes, including one of the variants (p.Ala311Thr) found in participant 1 [104]. This finding was later supported in two studies [102,105]. The overlap between an SLI phenotype and dyslexia is particularly interesting, given the extensive comorbidity (40% to 55%) reported for these two disorders [106]. The p.Ala311Thr, c.931G>A variant previously associated with SLI was also found to be a heterozygous deleterious variant in participants 9 and 10. This variant in these participants can therefore be reported as a VUS in CAS due to consideration of the allele frequency and functional and phenotypic data. On the basis of the comparison of the amino acid length, number of known polymorphisms and number of NS variants predicted to be damaging, this gene appears to be relatively highly conserved, in line with other known causal genes. A number of other *KIAA0319* variants were seen in the present participants, but all were deemed to be benign. None remained under consideration after application of the six prioritization guidelines.

### ATP13A4

Participant 1 has a heterozygous, relatively rare (estimated frequencies between 0.056 and 0.088, depending on the database queried), highly conserved variant (rs35424709) that gives rise to a NS amino acid change in *ATP13A4* (g.1938A>T, p.Glu646Asp). Despite this somewhat higher frequency, this variant was prioritized for consideration because this gene was previously causally associated with an overlapping phenotype.

*ATP13A4* is the least studied member of the subfamily of P5-type ATPases but has recently been postulated to play a vital role in the developing nervous system in early neuronal development [107]. The variant is located close to the conserved phosphorylation site (D468) and the nucleotide-binding domain of the protein. *ATP13A4* is highly expressed in areas of the brain known to be responsible for language, including the lateral inferior frontal cortex (Broca’s area) and the temporoparietal cortex (Wernicke’s area) [108]. Based on comparison of the amino acid length, number of known polymorphisms and number of NS variants predicted to be damaging, this gene appears to be relatively highly conserved, in line with other known causal genes. Although some damage prediction tools predict this change to be deleterious (Mutation Assessor, Condel, PolyPhen-2), others predict a neutral effect (PROVEAN, PolyPhen-2). An existing study of six individuals with autism spectrum disorder identified this as a deleterious change [108]. No phenotypic information is provided for the six study participants, so it is not possible to determine whether they have SLI or CAS phenotypes that could be associated with this variant in addition to their autism spectrum classification. Although language impairment is a major component of autism, individuals with autism may have a subtype of language disorder that differs from the type associated with CAS [109]. Thus, this study provides some support for a deleterious annotation for this variant, but the lack of participant phenotype information precludes speculation on causal pathways.

Notably, *ATP13A4* has previously been causally associated with CAS. A paracentric inversion disrupting the gene was identified in a seven-year-old girl with clinically defined expressive and receptive language delay [108]. The same inversion was found in the girl’s father, who also had language impairment. Expression analysis showed that the inversion resulted in inactivation of one copy of *ATP13A4* in each individual. Furthermore, one study recently provided functional evidence of the deleterious impact of the Glu646Asp variant in a mouse model [107]. Expression analysis of the wild-type and Glu628Asp (Glu646Asp orthologous equivalent) protein variant showed that the variant has no intrinsic calcium pump activity. The variant was shown to impair *ATP13A4* regulation of calcium transport to a significantly higher degree than that seen in alteration of the known phosphorylation site (D468Val) [107].

The *ATP13A4* variant was also found in two other participants in the present study: participants 2 and 4 (Table 4). Thus, three participants with CAS have the same variant, each in a heterozygous state. The existing functional evidence for a deleterious impact and the existing CAS associations in three unrelated participants with the same variants is interpreted as providing strong support for a causative role of the *ATP13A4* variant in CAS. The allele frequency is higher than expected, however, based on the rarity of this condition. Because of the latter and despite all of the functional and case study evidence in existence, we report this *ATP13A4* variant as a VUS in the present study for CAS in these three participants.

## Conclusions

The two goals of the present study were (1) to identify genomic variants in participants with CAS supporting literature trends indicating genetic heterogeneity and (2) to evaluate the productivity of WES for genomic studies in this and other pediatric motor speech disorders.

Regarding the first study goal, our findings support likely causal pathogenic mutations in two participants and additional clinically reportable variants in six of the remaining eight participants. A total of three participants had prioritized variants in two of six widely studied genes on five chromosomes. Participant 1 had noted variants in both *ATP13A4* and *KIAA0319*. Participant 4 had noted variants in *ATP13A4* and *CNTNAP2*. Participant 10 had noted variants in *KIAA0319* and *CNTNAP2*. In each of the three participants, the two genes reviewed are expressed in early neuronal development. These findings are consistent with the possibility of a two-hit causal mechanism, with interaction between genes or association within tightly regulated networks underlying participants’ behavioral phenotypes. Multiple-hit mechanisms have been posited in studies of autism and other neurodevelopmental syndromes [110,111].

With regard to the second study goal, the establishment of disease pathogenicity using next-generation sequencing remains controversial. For more than a decade, the prevailing perspective has been that implicating a causal gene or variant requires statistical confidence that there is less than a 1 in 1 million probability of finding the association by chance [112]. Without the availability of genome data from family members, we understood that our study would not be adequately powered to document new CAS genes. Rather, the second study goal was to evaluate the potential productivity of next-generation sequencing in CAS and other pediatric motor speech disorders.

We conclude that our findings demonstrate the potential productivity of next-generation sequencing in larger-scale genetic studies of simplex cases of CAS and possibly other motor speech disorders. In the present study, in which we used WES, we found a 60% success rate in detecting likely causal gene variants in the 10 present participants, compared to recent anecdotal estimates of 25% to 30% success rates in WES studies of other complex neurodevelopmental disorders. Crucially, the difference may reflect the strict inclusionary criteria for the disorder targeted in this study. Given that clinical exome sequencing currently costs less than $5,000 per patient, the cost of Sanger sequencing of all six target genes is now significantly in excess of whole-exome sequencing. Furthermore, the current list price for single-gene testing for *FOXP2* is over $2,250. In this situation, the cost per diagnosis made is significantly lower if whole-exome sequencing is performed ($8,333 per diagnosis assuming a 60% diagnosis rate and $16,666 assuming a 30% diagnosis rate) rather than single-gene testing on *FOXP2* ($56,250 per diagnosis assuming a 4% diagnosis rate). For well-phenotyped participants, next-generation sequencing of pediatric motor speech disorders likely may be a cost- and time-effective method of choice for both disease gene variant discovery and for clinical applications.

## Abbreviations

AOA2: Ataxia and oculomotor apraxia type 2; ASHA: American Speech-Language-Hearing Association; ASD: Autism spectrum disorder; CAS: Childhood apraxia of speech; GUS: Gene of uncertain significance; MSAP: Madison Speech Assessment Protocol; NGS: Next-generation sequencing; NS: Nonsynonymous; RNAi: RNA interference; SLI: Specific language impairment; SNV: Single-nucleotide variant; VUS: Variant of uncertain significance; WES: Whole-exome sequencing.

## Competing interests

The authors declare that they have no competing interests.

## Authors’ contributions

EAS and KJJ recruited and assessed participants and provided input on the manuscript. GR and JJL designed and directed the sequencing and provided input on the manuscript. BMW and JMH developed the analytical tools and assisted EAW with the bioinformatics analyses. DPD provided medical genetics input on the manuscript. EAW designed and completed the bioinformatics analyses and cowrote the manuscript. LDS designed and supervised the behavioral data collection and cowrote the manuscript. All authors read and approved the final manuscript.
